# How do antidepressants influence the BOLD signal in the developing brain?

**DOI:** 10.1016/j.dcn.2016.12.003

**Published:** 2016-12-21

**Authors:** Julia J. Harris, Clare Reynell

**Affiliations:** a Life Sciences Department, Imperial College London, SW7 2AZ, UK; b Francis Crick Institute, Midland Road, London, NW1 1AT, UK; c Département de Neurosciences, Université de Montréal, H3C 3J7, Canada

**Keywords:** BOLD fMRI, Depression, Antidepressant, Adolescence, Neurovascular coupling, Energy

## Abstract

Depression is a highly prevalent life-threatening disorder, with its first onset commonly occurring during adolescence. Adolescent depression is increasingly being treated with antidepressants, such as fluoxetine. The use of medication during this sensitive period of physiological and cognitive brain development produces neurobiological changes, some of which may outlast the course of treatment. In this review, we look at how antidepressant treatment in adolescence is likely to alter neurovascular coupling and brain energy use and how these changes, in turn, affect our ability to identify neuronal activity changes between participant groups. BOLD (blood oxygen level dependent) fMRI (functional magnetic resonance imaging), the method most commonly used to record brain activity in humans, is an indirect measure of neuronal activity. This means that between-group comparisons – adolescent versus adult, depressed versus healthy, medicated versus non-medicated – rely upon a stable relationship existing between neuronal activity and the BOLD response across these groups. We use data from animal studies to detail the ways in which fluoxetine may alter this relationship, and explore how these alterations may influence the interpretation of BOLD signal differences between groups that have been treated with fluoxetine and those that have not.

## 1 Introduction

From puberty onset to early adulthood, the brain undergoes significant structural (Ostby et al., 2009, Tamnes et al., 2010), physiological (Harris et al., 2011) and cognitive changes (Spear, 2000, Steinberg, 2005). This period of neurodevelopment is also associated with the highest lifetime risk of affective disorders, with the peak age of onset of any mental health disorder at 14 (Kessler et al., 2005, Paus et al., 2008). Major depression is the disorder with the highest lifetime prevalence (16.6%; Kessler et al., 2005), and its prevalence is three times higher in adolescence than in childhood (6% cf 2%, respectively; Costello et al., 2002). Thus, teenagers are particularly vulnerable to this debilitating and life-threatening disorder.

As major depression is becoming a leading cause of worldwide disability (Ferrari et al., 2013), and adolescents are increasingly recognised as a high-risk group, the prescription of antidepressant drugs to this population is rising – fast. A study this year found that, between 2005 and 2012, there was an average 41% increase in the number of young people prescribed antidepressants across the UK, US, Denmark, Germany and the Netherlands (Bachmann et al., 2016). These medications are being taken during a highly sensitive period of neurodevelopment, and recent evidence suggests that antidepressants may increase the risk of suicidal thoughts and behaviour in people younger than 25 (FDA, 2007, Le Noury et al., 2015). On the other hand, when regulatory warnings were put into place in the USA and Europe, the resultant reduction in antidepressant prescriptions was associated with an increased number of attempted and completed suicides in adolescents and young adults (Gibbons et al., 2006, Gibbons et al., 2007, Lu et al., 2014). It is therefore important to understand the effects of antidepressants on the brain, and particularly whether these effects are different between teenagers and adults (Cousins and Goodyear, 2015).

One way to investigate this is to use functional magnetic resonance imaging (fMRI) to measure how brain activity changes in response to antidepressant treatment in both adolescents and adults. Understanding how antidepressants affect the brain during mood-related tasks can also help elucidate the brain mechanisms of depression. However, for this approach to be most informative, it is essential to know how antidepressants affect all aspects of the signal that fMRI detects – the blood oxygen level dependent (BOLD) signal. In particular, any direct effects of antidepressants on neurovascular coupling or brain oxygen use could lead to errors in interpretation of BOLD data if not taken into account (see below). To help avoid such problems, we review here pharmacological and physiological research, from rodent to human, to provide a comprehensive summary of how antidepressants may affect neurovascular coupling and metabolism in the brain. We focus on the adolescent brain, and therefore on the antidepressant that is most commonly prescribed to adolescents, fluoxetine (also called Prozac).

## 2 The BOLD signal

Because of its non-invasive nature, fMRI is regularly used to study neuronal activity in humans. In disorders such as depression, it is an exceptionally useful tool, because mental health is particularly challenging to study using animal models. Importantly, however, the BOLD signal is not a direct measure of neural activity, but instead reports local changes in the amount of deoxyhaemoglobin in the blood, which are mediated by several different brain processes (Attwell and Iadecola, 2002). This topic has been comprehensively reviewed elsewhere (Attwell and Iadecola, 2002, Harris et al., 2011), so here we are brief. When neurons become active, the level of deoxyhaemoglobin in the nearby blood initially increases as oxygen use increases, and this decreases the BOLD signal (because deoxyhaemoglobin is paramagnetic). Blood flow to the region then increases, over-compensating for the oxygen use, and the deoxyhaemoglobin level falls, thus increasing the BOLD signal. The overall amplitude of the BOLD signal is determined by the balance of oxygen use (which decreases the BOLD signal) and blood flow increase (which increases the BOLD signal).

The diverse set of mechanisms by which neural activity leads to changes in blood flow is termed neurovascular coupling, and involves neuronal- and glial-mediated release of several molecules which can increase or decrease blood vessel diameter (Attwell et al., 2010). If any component of a neurovascular coupling pathway is affected by antidepressants, the BOLD signal could be altered even in the absence of a difference in neural activity. Similarly, any difference in brain metabolism and oxygen use could directly alter the BOLD signal. In order to ascribe between-group differences in the BOLD signal to differences in neural activity, it is important to be able to assume that the same set of processes is linking neural activity to oxygen use and blood flow in each group (Harris et al., 2011, Reynell and Harris, 2013). Is this a safe assumption in the case of antidepressants? We turn to this question after briefly reviewing what is known about the neurophysiology of depression and the pharmacology of antidepressants (Section 3), and then looking at some of the ways that fMRI is being used to study the effects of antidepressants on the adolescent brain (Section 4).

## 3 Depression and antidepressant treatment in adolescents

As mentioned above, major depression is a highly prevalent lifetime disorder, with its onset commonly occurring during adolescence. It is considered an episodic illness, although depression in adolescence is often associated with a chronic course, persisting into adulthood (Mueller et al., 1999, Solomon et al., 2000, Dunn and Goodyer, 2006). It is still unclear what causes depression, although we are increasingly aware of risk factors, such as early adverse experiences and childhood anxiety (Reinherz et al., 2003, Beesdo et al., 2007), substance abuse (Currie et al., 2005), and genetics (Sanders et al., 1999). Over the past several decades, research involving human patients and animal models has helped to build a picture of the neurobiological basis of depression (reviewed comprehensively in Nestler et al., 2002, Maletic et al., 2007). Our knowledge is far from complete, but many lines of evidence point towards structural and functional abnormalities in several brain areas including the prefrontal cortex, cingulate cortex, amygdala, striatum, hippocampus, hypothalamus and thalamus (Nestler et al., 2002, Maletic et al., 2007). Perhaps the most well known neuropathological aspect of depression is the “chemical imbalance” in the depressed brain. Depression is associated with impairments in neurotransmission, particularly in the transmission of monoamines including, dopamine, noradrenaline and serotonin (Nestler et al., 2002, Maletic et al., 2007), although the exact ways in which these neurotransmitters contribute to depressive episodes is still under debate (see Dunlop and Nemeroff, 2007, Cowen, 2008, Cowen and Browning, 2015). Interestingly, monoaminergic systems undergo significant functional changes over adolescence (Goldman-Rakic and Brown, 1982; reviewed in Harris et al., 2011). Augmenting the function of aminergic signalling is one of the most effective ways to treat the symptoms of depression.

The most common antidepressants are selective serotonin reuptake inhibitors (SSRIs) and, of this diverse class, only one is officially recommended for use in adolescents in the UK: fluoxetine (NHS guidelines, 2014; NICE guidelines, 2015). In practice, many adolescents are prescribed other SSRIs “off-label”, such as sertraline and citalopram, particularly in cases where adolescents do not respond well to fluoxetine. Recent meta-analyses show, however, that fluoxetine most effectively treats depressive symptoms in children and adolescents, with fewer adverse side-effects than other pharmacological antidepressant treatments (Hetrick et al., 2010, Cipriani et al., 2016). Fluoxetine is also prescribed as a treatment for other affective and anxiety disorders that show adolescent onset, including obsessive compulsive disorder, panic disorder, and bulimia nervosa (Hoffman and Mathew, 2008). Like other SSRIs, fluoxetine is thought to produce its antidepressant effect primarily by increasing the extracellular concentration of serotonin in the brain. Fluoxetine does this by blocking the reuptake of serotonin into cells by selectively binding to the serotonin transporter (SERT; Stahl, 1998). Several studies have shown that fluoxetine can also interact with serotonin receptors, in both agonistic (Peng et al., 2014) and antagonistic (Palvimaki et al., 1996) capacities.

As we will explore later, however, fluoxetine does not modulate serotonin function exclusively; it has several other neuropharmacological effects, for instance altering calcium signalling and astrocytic connectivity (discussed in Section 5). Because we do not fully understand the neuropathology of depression, we do not know which of the pharmacological effects of fluoxetine are the most important for relieving the symptoms of depression. Nor do we have a complete picture of how fluoxetine might alter brain-wide activity in the short-term or neurobiology in the long-term. fMRI is a potentially powerful tool with which to investigate these questions in patient populations.

## 4 Probing antidepressant effects on the developing brain using fMRI

Depression is fundamentally associated with disrupted emotional processing, which is thought to reflect a negative bias in how patients interpret external information (Beck, 1979, Teasdale, 1983, Watkins et al., 1996). One well-studied aspect of this is an impaired ability to interpret facial emotions (see meta-analysis by Dalili et al., 2015), which is commonly associated with abnormal activity in limbic brain regions. When viewing sad, fearful or angry faces, depressed adults show increased BOLD signals in the amygdala (Sheline et al., 2001, Victor et al., 2010, Zhong et al., 2011) and insular cortex (Keedwell et al., 2010, Zhong et al., 2011) compared to healthy adults. Interestingly, such increases in the BOLD response to negative stimuli can be reduced down to normal levels in depressed adults after treatment with various antidepressants (sertraline: Sheline et al., 2001; venlafaxine: Davidson et al., 2003), including fluoxetine (Fu et al., 2004), which suggests that these drugs act to decrease the negative bias that characterises depressive states (Warren et al., 2015).

Recently, it was shown that depressed adolescents have similarly augmented BOLD activation in limbic regions, particularly the amygdala, when viewing faces with negative emotions, compared to controls (Beesdo et al., 2009, Yang et al., 2010, Hall et al., 2014). Do these brain activity differences also show normalisation with antidepressant treatment? Tao et al. (2012) were the first to address this question. In non-medicated depressed adolescents compared to healthy controls, they found that fearful faces triggered greater BOLD responses in limbic brain regions (amygdala, orbitofrontal cortex and subgenual anterior cingulate cortex). Depressed adolescents were then started on a daily course of treatment with fluoxetine, and both groups of participants were scanned again 8 weeks later. This time, BOLD responses to fearful faces were no longer larger in the depressed group when compared to the control group, suggesting that, like in adults (Fu et al., 2004) fluoxetine treatment can “normalise” brain activity in response to negative facial expressions.

Thus, for limbic brain regions, the data from adolescent and adult studies are well aligned. In frontal brain regions, however, BOLD signal differences between depressed and healthy individuals may not be similar in adolescents and adults. In particular, adult studies tend to report a depression-related decrease in frontal activation compared to controls (Siegle et al., 2007, Keedwell et al., 2010, Zhong et al., 2011), whereas the findings in adolescents are less consistent. For instance, in non-medicated depressed adolescents compared to controls, Halari et al. (2009) report a pattern of decreased activation in prefrontal areas (such as the right dorsolateral prefrontal cortex and the inferior prefrontal cortex), whereas Tao et al. (2012) found increased frontal activation. There are several possible reasons for such experimental discrepancies, ranging from the statistical methods used for neuroimaging analysis to the complex developmental trajectory of the prefrontal cortex, which can itself be subdivided into multiple brain regions performing different functions. Nonetheless, fluoxetine appears to have a similar effect on frontal activation in both age groups. Tao et al. (2012) found that fluoxetine treatment in depressed adolescents decreased frontal activation to fearful faces, and Fu et al. (2004) found that fluoxetine treatment in depressed adults decreased frontal activation to the faces with the lowest affective load (ultimately increasing the dynamic range available for differential frontal activation by the most intensely sad faces).

It is an over-simplification to consider limbic and frontal brain regions entirely separately, as there is significant cross-talk between these areas through fronto-limbic connections. Just this year, Cullen et al. (2016) used fMRI to examine the effect of SSRIs, including fluoxetine, on fronto-limbic functional connectivity in depressed adolescents. They found that clinical response to treatment was associated with increased resting-state functional connectivity between amygdala and right frontal cortex, but decreased resting-state functional connectivity between amygdala and right precuneus and right posterior cingulate cortex.

**Fig. 1:**
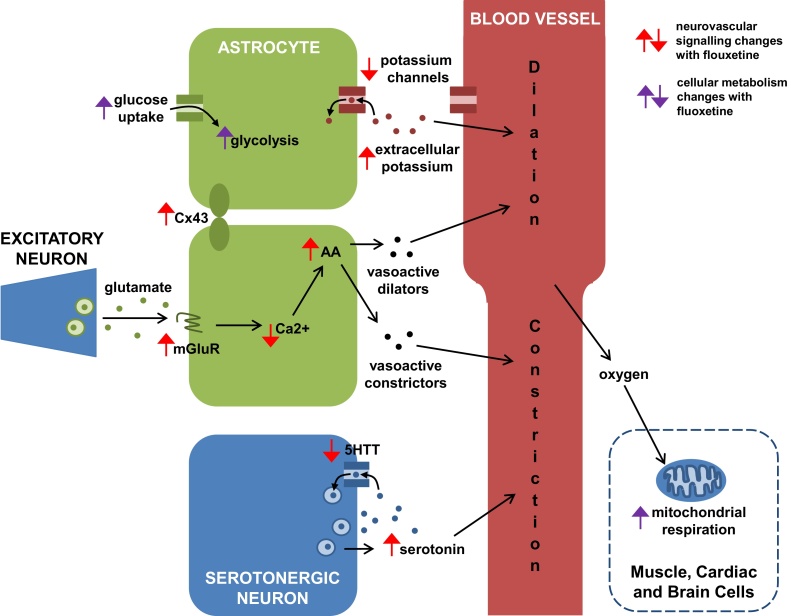
Pathways from neurons and astrocytes that regulate blood flow, leading to either dilation (black arrows to upper half of blood vessel) or constriction (black arrows to lower half of blood vessel) of the nearby vasculature. Red arrows indicate how points in these neurovascular signalling pathways are altered with fluoxetine treatment. Purple arrows describe how fluoxetine alters cellular metabolism. On the left, a synapse from an excitatory neuron releases glutamate onto an astrocyte. Glutamatergic activation of astrocytic metabotropic glutamate receptors (mGluRs) leads to increased intracellular calcium concentration and arachidonic acid (AA) production. AA is broken down into different vasoactive molecules, which can either dilate or constrict nearby blood vessels. Astrocytes are electrically connected via gap junctions made up of connexins, including connexin-43 (Cx43). Extracellular potassium (top) relaxes blood vessels, while serotonin release (bottom) provides a basal constriction of blood vessels. Serotonin transporters (5HTT) are the primary pharmacological target of SSRI antidepressants. Oxygen from the blood is used by all cells to produce energy via mitochondrial respiration. (For interpretation of the references to colour in this figure legend, the reader is referred to the web version of this article.)

Overall, fMRI data reveals both similarities and differences in the ways that adolescent and adult brains respond to depression and to antidepressant treatments. In order to accurately interpret BOLD signal differences between healthy and depressed groups, medicated and non-medicated groups, and adolescent and adult groups, it is essential to know whether the BOLD signal is reflecting the same set of brain processes between the groups. To this end, we now investigate the ways in which fluoxetine treatment is likely to affect the relationship between neural activity, blood flow response and cellular metabolism (summarised in Fig. 1).

## 5 Antidepressants and neurovascular coupling

### 5.1 Blood supply

The neurotransmitter that has been most commonly linked with depression is serotonin, which is a known vasoconstrictor and is responsible for setting vessel tone (Cohen et al., 1996). Although the vasoconstricting role of serotonin is widely accepted, there is some evidence that it also can also have a dilatory action (Edvinsson et al., 1977). These opposing effects of serotonin are thought to be due to differing expression of adrenergic receptors (Mylecharane, 1990). Whether serotonin is vasodilating or constricting, it is clear that any change in its concentration which may underpin changes in mood, could also lead to changes in blood vessel tone. Such changes in basal vessel tone will, in turn, alter the vascular response to neuronal activity (Blanco et al., 2008). Interestingly, a study measuring baseline cerebral blood flow in adolescents recorded both regions of increased blood flow and regions of decreased blood flow in participants with depression, compared to healthy controls. Significant hypoperfusion was recorded in executive, affective and motor networks (frontal, amygdalar, insular, cingular and cerebellar regions) while significant hyperperfusion was recorded in areas supporting emotional regulation (subcallosal cingulate, putamen, and fusiform gyri; Ho et al., 2013). Differences in cerebral blood flow are traditionally interpreted to reflect metabolic abnormalities (see Section 6) and altered neuronal activity in depressed individuals. However, changes in blood flow could also be due to abnormal vessel tone, which would alter vessel responsivity – vessels that are either too dilated or constricted in baseline states could have impaired haemodynamic responses to neuronal activity. Interestingly, most antidepressants, including fluoxetine, increase extracellular serotonin concentration. As well as affecting neuronal activity, this increase in serotonin could directly alter vessel tone, perhaps with a therapeutic effect on the relationship between neuronal activity and blood flow response.

*In vitro* studies of isolated rat cardiac and cerebral arterioles found that acute fluoxetine application causes a concentration-dependent vessel dilation and reduced constriction in response to serotonin and noradrenaline (Pacher et al., 1999, Ungvari et al., 1999). Similar results were found in experiments carried out in isolated skeletal muscle arterioles. Here, the decrease in constriction was due to fluoxetine-induced inhibition of calcium entry to muscle cells through L-type calcium channels (Ungvari et al., 2000). *In vivo* recordings in rats have also found dilation of cerebral arterioles upon acute fluoxetine administration (Ofek et al., 2012), and these responses were measured alongside an increase in calcium in endothelial cells. Investigations into the effect of chronic fluoxetine treatment (21 days) have, however, found opposing results. An *in vivo* study found that chronic fluoxetine causes mild hypertension in rats, suggesting increased vessel constriction, along with decreased vessel responsivity to vasodilators (Crestani et al., 2011). However, more recent work found evidence contradicting this interpretation. Pereira et al. (2015) showed that mesenteric arteries that had been dissected from rats treated with fluoxetine for the previous 21 days displayed *increased* vessel responsivity to vasodilators, compared to mesenteric arteries from untreated rats. The authors suggest that this increased dilatory response was due to upregulated endothelial nitric oxide production and activation of calcium sensitive potassium channels (Pereira et al., 2015).

There is more than one possible reason for the different conclusions drawn from the studies of Crestani et al. (2011) and Pereira et al. (2015). Crestani et al.’s work was carried out *in vivo*, examining the cardiovascular system, whereas Pereira et al. focused on the mesenteric blood supply, and examined the effects of vasodilators directly on these vessels *in vitro*. Although studies of chronic drug treatment have the advantage of being more comparable to the long-term administration of antidepressants in humans, the results can be complicated by the development of indirect, sometimes compensatory, physiological adaptations. It will therefore be important to continue employing both acute and chronic treatment approaches in different model systems to build a full picture of how fluoxetine directly affects the blood supply in the brain and how, in turn, it may affect the BOLD signal.

### 5.2 Astrocyte morphology and connectivity

Astrocytes, a type of glial cell in the brain, are an important component of neurovascular coupling as they are able to sense neuronal activity and respond by signalling an increase in energy use and demand to the vasculature (for a more detailed description of this process see Attwell et al., 2010, Harris et al., 2011). Two key features that allow astrocytes to play this role are (1) the coverage of blood vessels by astrocyte endfeet and (2) the connectivity between single astrocytes to form a glial network, which allows for the rapid spread of signals through specialised “gap junctions” made up of proteins called connexins (Giaume and McCarthy, 1996).

Depression is thought to be linked to several glial abnormalities. In post-mortem studies of patients with depression, astrocytes have been reported to have decreased coverage (Rajkowska et al., 2013, Sun et al., 2012) and connectivity (Miguel-Hidalgo et al., 2014, Sun et al., 2012). This decrease in connectivity has been demonstrated by a reduction in connexin expression in post-mortem brains of depressed patients (Miguel-Hidalgo et al., 2014) as well as by decreased spreading of gap-junction permeable dyes between astrocytes in an animal model of depression induced by chronic stress (Sun et al., 2012).

Many studies suggest that a variety of antidepressants affect astrocytic function, and these are well reviewed by Czeh and Di Benedetto (2013). Short-term application (48 h) of fluoxetine to cultured astrocytes increased the number and complexity of astrocyte processes in cultures derived from rats displaying either normal or high anxiety behaviour (Di Benedetto et al., 2016). This increase in astrocyte plasticity was, however, no longer observed if aquaporin-4 production was inhibited. This is of note due to the decreased aquaporin-4 expression measured alongside decreased astrocyte endfeet coverage in human postmortem brains of MDD patients (Rajkowska et al., 2013). *In vivo*, however, 48 h of fluoxetine injection did not increase astrocyte coverage of blood vessels in either normal or high anxiety rats (Di Benedetto et al., 2016). These results suggest that a longer treatment period may be necessary to fully restore astrocyte morphology, even though short-term treatment can increase astrocyte plasticity, perhaps sufficiently to strengthen the connection between neuronal activity and vascular response. Short-term (24 h *in vitro*: Mostafavi et al., 2014) and long-term (21 days *in vivo*: Fatemi et al., 2008) treatment with fluoxetine has also been found to increase the expression of connexin-43, the gap-junction that forms connections between astrocytes. Sun et al. (2012) replicated the increase in connexion-43 seen with 21 days of fluoxetine treatment *in vivo* and showed that it leads to increased functional connectivity between astrocytes as measured by the spread of a gap-junction permeable dye. Such increased astrocytic connectivity could allow for better signal transmission throughout a larger glial network. It should be noted, however, that increased connexin expression was seen after fluoxetine treatment in rats that were under chronic stress, but not in control rats, and the effect may therefore be specific to this animal model of depression.

### 5.3 Signalling pathways

The signalling pathways involved in neurovascular coupling (reviewed in detail in Howarth, 2014) involve both neurons and astrocytes. These pathways include activation of ionotropic and metabotropic glutamate receptors, calcium signalling, potassium signalling, and the production of nitric oxide and several vasoactive metabolites of arachidonic acid (AA; Attwell et al., 2010, Harris et al., 2011, Howarth, 2014). Here we will explore the alterations in these pathways that have been observed in depression, and investigate their response to antidepressant treatment.

#### 5.3.1 Glutamate receptors

Metabotropic glutamate receptors are thought to play an important role in the astrocytic signalling pathways of neurovascular coupling (Attwell et al., 2010). Both increases and decreases in metabotropic receptor expression have been recorded in depression. A decrease in group 1 metabotropic glutamate receptor (mGluR5) expression was found in postmortem brains of humans with depression (Deschwanden et al., 2011) whilst similar studies have found an increased expression in group 2 metabotropic glutamate receptors (mGluR2/3, Feyissa et al., 2010). Curiously, animal studies using western blot analysis of several brain regions including the cerebral cortex, hippocampus and corpus striatum show that although acute treatment with imipramine, a tricyclic antidepressant, causes a reduction in group 2 receptor expression, chronic treatment leads to the upregulation of these receptors (Matrisciano et al., 2002). In an animal model of depression, induced by early-life stress through maternal separation, there is a decrease in the expression of group 3 metabotropic glutamate receptors (mGluR4) in the hippocampus which recovers to control levels after chronic fluoxetine treatment (O’Connor et al., 2013).

Ionotropic glutamate receptors, which are involved in the neuronal signalling pathways of neurovascular coupling, are also altered in depression. One post-mortem study found a decrease in the expression of NMDA receptor subtypes, NR2A and NR2B, in the prefrontal cortex of depressed patients (Feyissa et al., 2009). Interestingly, chronic fluoxetine also decreases the expression of these NMDA receptor subtypes (NR2A and NR2B along with NR1) in the hippocampus and prefrontal cortex of mice (Stan et al., 2015). Conversely, Ampuero et al. (2010) found that chronic fluoxetine leads to an increase in expression of NMDA receptor subtype NR2A in the cerebral cortex. This study, along with others, also found that fluoxetine alters the subunit expression of AMPA-kainate receptors in an area-specific manner, showing both increased and decreased expression in hippocampus and prefrontal cortex (Barbon et al., 2006, Ampuero et al., 2010). These changes in subunit expression, and thus receptor stoichiometry, of glutamate receptors are supported by changes in radioligand binding at NMDA receptors following chronic fluoxetine treatment (Nowak et al., 1998). Phosphorylation of both AMPA and NMDA receptor subunits have also been found to be increased upon fluoxetine treatment (Svenningsson et al., 2002, Stan et al., 2015), which could contribute to changes in receptor function. A study investigating the effect of acute fluoxetine application on glutamate receptor behaviour in cultured neurons found that both ionotropic and metabotropic glutamate receptors showed decreased calcium responses following treatment (Kim et al., 2013).

Changes in glutamatergic transmission in depression, and during fluoxetine treatment, would be expected to have several effects on downstream signalling, including the signalling pathways involved in neurovascular coupling.

#### 5.3.2 Calcium

Calcium signalling has long been thought to be altered in depression (Jimerson et al., 1979, Bowden et al., 1988). Acute fluoxetine application has been found to produce calcium responses independently from neuronal activity in a significant number of astrocytes both in cell culture (Li et al., 2011a) and in acute brain slices (Schipke et al., 2011). Chronic treatment with fluoxetine leads to a change in astrocytic calcium homeostasis resulting from a decrease in the calcium release from intracellular stores upon astrocyte receptor stimulation with agonists (Li et al., 2011a). This is thought to be caused by a decreased calcium conductance through ion channels (TRPC1 and GluK2), resulting in an insufficient calcium store to produce responses on agonist application (Li et al., 2011a, Li et al., 2011b). This may mean that, during fluoxetine treatment, astrocytes are not able to produce an equivalent calcium signal to that observed in control cells in response to neuronal activity.

#### 5.3.3 Nitric oxide

Nitric oxide is a vasodilator that plays a role in the neuronal signalling pathway of neurovascular coupling. Glutamate released during neuronal activity increases nitric oxide production as a result of NMDA receptor activation (Attwell et al., 2010). In a depression-mimicking animal model, nitric oxide synthase levels are increased compared to control levels (Luo and Tan, 2001). Fluoxetine treatment, in this model of depression, reduces nitric oxide synthase expression to levels similar to those found in control animals (Luo and Tan, 2001). Additionally, several studies have found that fluoxetine decreases the NMDA receptor-mediated nitric oxide response (Li et al., 2006, Crespi, 2010). Together, these results suggest that fluoxetine inhibits the neuronal signalling pathway that leads to nitric oxide-dependent dilation of the vasculature. Direct inhibition of nitric oxide production has been investigated as a possible treatment for depression. Treatment with the specific nitric oxide synthase inhibitor, 7-NI, prevents the induction of cardiovascular changes and depression-like behaviours seen in animals exposed to a chronic stress protocol (Almeida et al., 2015). It is unclear whether the antidepressant effects of nitric oxide inhibition are produced through the same mechanism as SSRIs. Support for a common mechanism of action comes from Harkin and colleagues, who suggest that the antidepressant effects of nitric oxide synthase inhibitors are dependent on endogenous serotonin, as they are blocked by serotonin depletion (Harkin et al., 2003). Conversely, the same lab also found that nitric oxide synthase inhibition augments the effect of fluoxetine which would suggest the mechanism of action was independent (Harkin et al., 2004).

The decrease in nitric oxide signalling observed in the presence of fluoxetine suggests that the dilatory signal produced through the neuronal pathway of neurovascular coupling would be weaker during antidepressant treatment, and thus blood flow responses to the same level of neuronal activity may be reduced.

#### 5.3.4 Arachidonic acid

Calcium signalling in astrocytes leads to the production and breakdown of arachidonic acid into its vasoactive metabolites including prostaglandin, EETs and 20-HETE (Attwell et al., 2010). Several studies have found that chronic fluoxetine treatment increases arachidonic acid production and breakdown (Lee et al., 2007, Li et al., 2009). These data are unexpected as a decrease in calcium signalling would predict a decrease in arachidonic acid production. Regardless of whether these changes agree with those in the previous section, it appears likely that fluoxetine treatment would lead to a change in the production of vasoactive arachidonic acid derivatives.

#### 5.3.5 Potassium

Another astrocytic signalling pathway downstream of calcium signaling is the modulation of extracellular potassium which produces a change in the tone of the smooth muscle surrounding arterioles. Astrocytes are relatively leaky cells and this allows them to influence the extracellular potassium concentration. This process is termed potassium buffering. Increased extracellular potassium leads to smooth muscle hyperpolarization and relaxation (Filosa et al., 2006), which dilates the arteriole. There are several links between potassium channel expression and depression. Different genetic variants of the two-pore domain potassium channel TREK-1 (encoded by the KCNK2 gene), which contributes to setting the resting membrane potential, are linked to mental illness (Congiu et al., 2015). There is evidence that deletion of KCNK2 in mice leads to a depression-resistant animal model (Heurteaux et al., 2006). Additionally, in this study, fluoxetine was shown to inhibit TREK-1, supporting the same finding from previous work by Kennard et al. (2005). There is also evidence that fluoxetine inhibits inward rectifying potassium channels in cultured astrocytes (Ohno et al., 2007, Furutani et al., 2009). This change would lead to impaired potassium buffering in astrocytes and thus increased extracellular potassium. Other voltage-gated potassium channels have also been found to be inhibited by fluoxetine in other cell types (Choi et al., 1999, Thomas et al., 2002). Any changes in potassium buffering and conductance would alter the role of potassium in neurovascular signalling.

### 5.4 Fluoxetine-induced neurovascular coupling changes and the BOLD signal

Any of these fluoxetine-induced changes in neurovascular coupling could lead to a change in the BOLD signal response to neuronal activity. One could imagine, for example, that a fluoxetine-induced increase in vessel tone (Crestani et al., 2011) would mean that a stronger signal would be required to produce an increase in blood flow (Blanco et al., 2008), thus the same amount of neuronal activity would lead to a smaller change in the BOLD signal. Fluoxetine-induced increases in astrocyte complexity and connectivity (Di Benedetto et al., 2016) could mean that a larger glial area is able to respond to neural activity and communicate to the vasculature, thus the blood flow response would be seen over a larger region. On the other hand, if fluoxetine decreases the calcium storage and conductance in astrocytes (Li et al., 2011a, Li et al., 2011b), the effectiveness of astrocytic communication to the vasculature would be decreased and thus a weaker blood flow response to the same neural activity could be seen with fluoxetine treatment compared to control. Clearly, there are several ways in which fluoxetine is likely to produce neurovascular coupling changes, thus directly affecting the BOLD signal. Because we cannot currently be sure of the extent – or even the direction – of this influence, further research into the effects of fluoxetine on neurovascular coupling is critical.

Most of the animal studies in this section were carried out at ages that would correspond to human adulthood. Unfortunately, to date there has been little study of the specific effects of fluoxetine on neurovascular coupling during adolescence, and it is not easy to extrapolate the effects from adult studies, because several changes to the vasculature and neurovascular coupling pathways take place over this period. These changes are discussed in detail in our previous review (Harris et al., 2011), but we include some examples here, highlighting their relationship to depression and fluoxetine. For instance, over adolescence, there is a decrease in neuronal expression of the enzyme responsible for synthesising the vasodilator, nitric oxide. Interestingly, we have seen that endothelial production of nitric oxide is increased with fluoxetine treatment (Pereira et al., 2015). Adolescence is also associated with an increase in the expression of receptors for prostaglandins, which are a vasodilatory breakdown product of arachidonic acid. As discussed above, the production and breakdown of arachidonic acid is increased with fluoxetine treatment (Lee et al., 2007, Li et al., 2009). Finally, mGluR5 receptor expression decreases over adolescence, and we have seen that the expression of these receptors is also reduced in depression (Deschwanden et al., 2011). These neurovascular changes through adolescence suggest that some of the effects of fluoxetine may differ depending on the stage of development, making it important to build a complete picture of the many ways in which fluoxetine can directly affect components of neurovascular coupling at the cellular level. This is critical for improving our understanding of the ways that fluoxetine treatment could affect the BOLD signal without affecting neuronal activity itself, at different stages of life.

## 6 Antidepressants and brain energy use

### 6.1 Human studies of brain energy use in depression and with fluoxetine treatment

All brain cells use energy in the form of adenosine triphosphate (ATP), which is generated intracellularly via both anaerobic respiration using glucose (glycolysis) and aerobic respiration, which also requires oxygen (oxidative phosphorylation). In humans, brain energy use is often measured using fluorodeoxyglucose positron emission tomography (FDG-PET), which reports glucose uptake by cells, thus indicating brain regions of high glycolytic activity. Although it is not possible to distinguish which cell type is taking up glucose, this method is considered to provide insight into the brain-wide distribution of neuronal activity, since active neurons require more energy than inactive neurons (Sokoloff, 1981). Do the patterns of brain glucose uptake, as measured by FDG-PET, align with the patterns of increased blood flow, as measured by fMRI, in depressed patients and those treated with antidepressants?

FDG-PET studies in depressed adult humans typically reveal glucose hypometabolism in frontal brain regions and glucose hypermetabolism in limbic brain regions compared to controls, which mirrors the pattern of task-evoked BOLD signal abnormalities often seen in depression (discussed in Section 4, above). Treatment with different antidepressants has been suggested to “normalise” brain metabolism by both increasing (Kennedy et al., 2001) and decreasing (Kennedy et al., 2001, Drevets et al., 2002) glucose uptake, depending on the brain region.

The specific effects of fluoxetine on brain glucose metabolism were studied by Mayberg et al. (2000), who carried out FDG-PET scans in depressed adults after 1 and 6 weeks of fluoxetine treatment. For several brain regions, the changes to baseline brain metabolism seen after 1 week of treatment were very different to those seen after 6 weeks of treatment, and it was the metabolic pattern at 6 weeks that correlated with behavioural response. Specifically, clinical improvement in behaviour was associated with limbic decreases and prefrontal increases in glucose uptake at 6 weeks of treatment. While seemingly in line for the limbic system, these metabolic changes could appear at odds with the finding that fluoxetine treatment decreases task-evoked BOLD responses in the frontal cortex, in both adults and adolescents as compared to controls (discussed above, Fu et al., 2004, Tao et al., 2012). However, it is important to remember that the metabolic changes observed in the PET study were not task-evoked, while the BOLD signal differences were observed in response to facial stimuli of negative emotional valence. Interestingly, antidepressants may affect the BOLD response of frontal sub-regions to positive and negative emotional stimuli differently (see meta-analysis by Ma, 2015). Thus, it is not straightforward to compare region-wide changes in baseline glucose metabolism to the task-evoked changes in specific subregions that are detected using fMRI.

Additionally, the BOLD signal does not directly reflect neuronal metabolism. Thus, increased brain metabolism could be associated with either an increased BOLD signal, if more active neurons signal to the vasculature, or a decreased BOLD signal, if the neurovascular response remains the same but more oxygen is used. Additionally, an increase in regional glucose uptake, as measured by FDG-PET, may not be directly related to neuronal activity – it could reflect increased glucose uptake by non-neuronal brain cells, and we cannot necessarily assume that the relationships between glycolysis, oxidative phosphorylation, ATP production, and ATP use are constant, particularly in disease states or in the presence of pharmacological treatment. It is therefore critical to know whether fluoxetine affects energy production pathways or energy use by cells directly. We now examine the effects of fluoxetine on glucose and oxygen metabolism at the cellular level.

### 6.2 Direct effects of fluoxetine on cellular metabolism

One possible reason for a fluoxetine-related increase in baseline glucose metabolism but not task-evoked BOLD response in the prefrontal cortex is that glucose uptake may be increased in astrocytes – not neurons. Depression has been associated with neuronal pathology in the prefrontal cortex (Rajkowska et al., 1999, Cotter et al., 2002), and one reason for this may be the loss of support from astrocytes, which are also damaged or lost in depression (Rajkowska et al., 1999, Cotter et al., 2002, Si et al., 2004, Choudary et al., 2005, Banasr and Duman, 2008). Allaman et al. (2011) found that fluoxetine application to cultured cortical astrocytes upregulated the astrocytic production of neuronal growth factors and increased glucose utilisation and lactate release. As astrocytes are thought to provide energy to neurons in the form of lactate (Pellerin and Magistretti, 1994) these results suggest that fluoxetine may facilitate the astrocytic provision of trophic and metabolic support to neurons, perhaps reducing depression-associated neuronal atrophy in the cortex. Interestingly, similar results were found with treatment using a different SSRI (paroxetine) but not with two tricyclic antidepressants (imipramine and desipramine). This implies that the mechanism is not simply through raised serotonin levels, but a direct effect of the SSRIs, fluoxetine and paroxetine, on astrocyte metabolism.

Shumake et al. (2010) studied the effects of fluoxetine on brain metabolism and behavioural performance in an animal model of depression, the congenitally helpless rat. They found that two weeks of fluoxetine treatment had positive effects on motivation, increasing climbing and reducing immobility on a forced swim test. These behavioural effects were associated with changes in cytochrome oxidase activity, a critical enzyme for aerobic respiration through oxidative phosphorylation by mitochondria. Cytochrome oxidase activity was decreased in the prefrontal cortex, suggesting that oxygen metabolism in the prefrontal cortex was reduced. This result is not necessarily at odds with the finding that glucose metabolism in the prefrontal cortex is increased (Mayberg et al., 2000), as glucose utilisation for glycolysis is not necessarily coupled to oxygen utilisation for oxidative phosphorylation. Perhaps, as suggested above, prefrontal glucose utilisation is increased in astrocytes, allowing trophic support and therefore survival of local neurons. But these neurons will not necessarily become more active − if fluoxetine treatment decreases the activity of neurons in the prefrontal cortex, then activity-related neuronal oxygen consumption will also decrease (Hall et al., 2012).

Nonetheless, fluoxetine might also have direct effects on cellular oxygen use, by altering mitochondrial respiration. Recent studies on rats suggest that exposure to fluoxetine during development results in increased aerobic respiration by mitochondria that persists in adulthood. Da Silva et al. (2015) treated rats with fluoxetine from birth until 21 days of age. At 60 days (which is considered adulthood in rat), they found increased mitochondrial respiration in both skeletal muscle and brain (hypothalamus). Along similar lines, Braz et al. (2016) found that mitochondrial respiratory capacity in cardiac tissue was increased by 23% in adult rats who had been treated with fluoxetine during development compared to rats who had not. This increased mitochondrial respiration – and therefore increased oxygen use – could be a general and long-term effect of fluoxetine treatment during development that is not related to neuronal activity.

### 6.3 Fluoxetine-induced cellular metabolism changes and the BOLD signal

Fluoxetine appears to have direct effects on both glucose use and oxygen use in the brain. Any changes in oxygen use will directly affect the BOLD signal. For instance, the fluoxetine-induced increase in mitochondrial capacity, as seen by Da Silva et al. (2015) and Braz et al. (2016), could mean that more oxygen is used even when neural activity and neurovascular signalling remain the same. This would lead to a smaller BOLD signal, which should not be interpreted as a decrease in neural activity. This idea is, of course, possible but speculative, and serves simply to highlight the fact that we cannot confidently parse the contribution of fluoxetine-induced metabolic changes and fluoxetine-induced neural activity changes to differences in the BOLD signal between groups. Before this is possible, more work is needed to fill in the gaps in our knowledge concerning how fluoxetine affects the relationships between cellular oxygen and glucose usage for ATP production, and ATP usage for neural activity.

Interestingly, abnormalities in brain metabolism are increasingly being associated with a wide range of neurodegenerative and neuropsychiatric disorders, including major depression (Berk et al., 2011, Siwek et al., 2013, Hroudová et al., 2014). Thus, some of the metabolic effects of fluoxetine should perhaps be viewed as therapeutic. In this vein, it is compelling to note that many of the metabolic effects of fluoxetine, such as increased astrocytic support of neurons or persistent augmentation of mitochondrial function, appear to be relatively slow-acting. As such, it seems possible that metabolic normalisation may account for some of the delayed benefits of treatment, perhaps explaining why depression symptoms are not alleviated as soon as serotonin levels in the brain are increased (e.g. Allaman et al., 2011). These long-term metabolic responses to treatment are therefore particularly relevant to the still-developing adolescent brain.

## 7 Long-term effects of antidepressants taken during adolescence

Anderson and Navalta (2004) put forward the hypothesis that, in adolescence, long-term effects of drugs may be delayed and only expressed once the vulnerable system reaches maturation (i.e adulthood). This phenomenon is referred to as neuronal imprinting and occurs when the effects of drug exposure outlast the drug itself.

The use of SSRIs in development has been shown to have an effect on behaviour in adult mice and rats (Ansorge et al., 2004, Iniguez et al., 2010). Iniguez et al. (2010) found that adolescent male rats treated with fluoxetine for 15 consecutive days (postnatal days 35–49) displayed increased anxiety and decreased copulatory behaviour three weeks after treatment. Conversely, rats that were treated during adulthood (fluoxetine administered on postnatal days 65–79), did not show long-lasting increases in anxiety.

Chronic fluoxetine treatment at different developmental stages also has different effects at the cellular level. In adult animals, fluoxetine treatment triggers either no change or a reduction in serotonin transporter expression (Lesch et al., 1993, Pineyro et al., 1994, Wegerer et al., 1999) whereas, in juvenile animals, fluoxetine treatment leads to a long-term increase in the expression of serotonin transporters in the frontal cortex (Wegerer et al., 1999). This result could reflect an increase in the density of serotonin transporters per synaptic terminal, or it could reflect an increase in the number of synaptic terminals themselves. The authors favour the latter theory, suggesting that fluoxetine treatment enhances the serotonin-triggered release of astrocytic growth factors, which in turn upregulate the growth of serotonergic nerve terminals, so long as the frontal cortex is still developing and relatively plastic. Other long-lasting cellular-level effects of fluoxetine treatment during adolescence include an increase in S100B, a glia-derived calcium-binding protein which may influence the development of serotonergic fibers (Bock et al., 2013) and, as discussed in Section 6, increased mitochondrial respiration (Da Silva et al., 2015, Braz et al., 2016).

The functional brain response to serotonin of adolescent- versus adult-treated rats is less clear. An acutely administered high dose of fluoxetine suddenly increases serotonin levels in the brain, and this “serotonin challenge” leads to an increased BOLD signal in several regions of the rat brain. Compared to untreated rats, this BOLD response to serotonin challenge is smaller when rats had previously been chronically treated with fluoxetine during adulthood (Klomp et al., 2012, Bouet et al., 2012). Chronic treatment with fluoxetine during adolescence has been observed to either increase (Klomp et al., 2012) or decrease (Bouet et al., 2012) the BOLD response to serotonin challenge, compared to untreated rats.

These findings support the idea that the adolescent brain is particularly vulnerable to pharmacological treatment, and that chronic fluoxetine treatment in adolescence may lead to changes in brain development that differ from the long-term adaptational changes that occur in the mature brain in response to the same treatment. This should be taken into account when studying adult participants who were treated with fluoxetine during adolescence. Many fMRI studies require participants to cease fluoxetine treatment prior to assessment, with a common exclusion criterion being treatment within the last four weeks. Sometimes this treatment-free period is longer or shorter, and occasionally it is not specified, with participants simply reported as “medication-free”. Considering the long-term effects that antidepressant drugs may have on neurovascular coupling and brain metabolism, particularly if the treatment was given during adolescence, it is not clear what period of cessation is sufficient to remove the neuronal, neurovascular coupling, or brain metabolic effects of fluoxetine on the BOLD signal. In clinical practice, patients are encouraged to come off SSRI medication slowly, gradually reducing the dose over weeks to months because severe psychological and somatic side effects are often observed when treatment is stopped abruptly (Black et al., 2000, Fava, 2006). The presence of withdrawal effects after stopping treatment would certainly imply that the physiological and pharmacological effects of the drug are not reversed within a short time frame.

## 8 Overcoming limitations

We have seen that fluoxetine treatment can have direct effects on neurovascular coupling pathways and cellular metabolism, which may last beyond the treatment period, especially if the drug was given during the sensitive period of adolescence. These wide-ranging neurophysiological effects of fluoxetine make it difficult to attribute BOLD signal differences between treated and untreated patient groups to differences in neural activity. Nevertheless, fMRI is one of the few tools that allows for non-invasive examination of brain activity in humans, and is therefore one of the most promising techniques with which to study the effects of drug treatment in depressed patients. Are there ways that we can overcome the interpretational complications caused by drug effects on neurovascular coupling brain energy use?

An ideal solution would be to include an experimental control task, which could eliminate the possibility of differences in neurovascular coupling and energy use contributing to BOLD signal differences (Iannetti and Wise, 2007). This approach has been explored by several groups (e.g. Kang et al., 2003, Murphy et al., 2009, Feczko et al., 2012), with the following archetypal format. In addition to the task of interest, the authors might study the BOLD signal response to a low-level visual task. If this task does not produce different BOLD responses in the occipital cortex between two participant groups, then it is assumed that there is no global difference in neurovascular coupling between these two groups, and it is argued that any BOLD signal differences that are observed during the task of interest can thus be attributed to neural activity differences. Unfortunately, as discussed in detail in our previous review (Reynell and Harris, 2013), there are problems with this line of reasoning. The issue is that neurovascular coupling mechanisms vary widely between different brain regions (Devonshire et al., 2012) and thus, any conclusions drawn from data obtained in one brain region during one task, cannot be safely extrapolated to any other brain region or any other task.

Unfortunately, extrapolations within one brain region or task are not safe either. If, for instance, there are between-group BOLD signal differences for one set of stimuli (e.g. fearful faces) – but not another set of stimuli (e.g. happy faces) – within the same task and brain region, one might be tempted to exclude the possibility that neurovascular coupling differences could be responsible. However, neurovascular coupling mechanisms can vary even within the same brain region when different neuronal inputs are activated (Enager et al., 2009). Because different stimuli will activate different neurons (particularly in higher-order processing regions), it is entirely possible that the neurovascular coupling mechanisms associated with one stimulus type could be affected differently to the neurovascular coupling mechanisms associated with another stimulus type, even within the same brain region. The major difficulty with any task-based approach to excluding neurovascular coupling confounds is that neurovascular coupling changes are not necessarily global: they can theoretically be just as task- and region-specific as neuronal differences themselves.

Particularly relevant for mood disorders, recent work has shown that the neuromodulator, dopamine, decreases visually-evoked BOLD signals at the same time as increasing the local cerebral blood flow, suggesting a disproportionate increase in energy metabolism (Zaldivar et al., 2014). Thus, as neuromodulator concentrations change throughout the day and during different behaviours (or with drug treatment), the relationships between the BOLD signal, blood flow, energy metabolism and neuronal activity can be profoundly altered. If these relationships are not constant across all brain regions and neuronal pathways, then the employment of a control task or stimulus will never be sufficient to exclude the possibility that neurovascular coupling differences are in fact responsible for BOLD signal differences seen with any other task, stimulus or brain region of interest.

The most promising approach for separating neuronal activity contributions to the BOLD signal from neurovascular coupling or metabolic contributions, is to directly measure electrical activity, using techniques such as EEG (Vitali et al., 2015), and/or blood flow, using techniques such as arterial spin labelling (Wang et al., 2011). Performed alongside fMRI, data from these techniques can reveal whether the relationship between neuronal activity and blood flow remains constant across participant groups, brain regions and behavioural tasks. The addition of even one of these techniques can vastly improve the interpretation of fMRI data. For example, if an increased BOLD response is accompanied by an increased EEG response to the same task, this is a good indication that the BOLD signal increase reflects an increase in neuronal activity. If, on the other hand, data from the two techniques contradict each other, this would indicate that additional factors, such as neurovascular coupling differences, are contributing to the BOLD signal difference between groups. Several labs and clinicians are beginning to implement direct measures of neural activity or blood flow alongside fMRI, with promising results (e.g. Dinstein et al., 2012; and see Wang et al., 2011 and Vitali et al., 2015 for thorough reviews).

For fMRI studies investigating the brain mechanisms of drug action in particular, it may be useful to employ some additional techniques to dissociate the pharmacological effects on neural activity and neurovascular coupling. Murphy and Mackay (2011) provide a thoughtful discussion of the promising directions and general caveats for fMRI analysis of drug action. One interesting approach is time-series analysis; examining BOLD signal changes during the precise time-scale over which the drug is known to act. If these changes correspond to the time-scale of behavioural changes, then it is likely that they reflect some changes in neuronal processing. This method involves acute administration of the drug, which has the advantage that a placebo control group can also be examined, without the ethical concerns of longer-term placebo treatment. A behavioural difference between the drug and placebo group (as has recently been observed for acute fluoxetine treatment; Capitão et al., 2015), would lend confidence to the idea that the drug causes neural activity differences, which could theoretically be detected by fMRI. Of course, whether the BOLD signal differences that are observed accurately represent these neural activity differences, still depends on whether the relationship between neural activity, blood flow and energy use is the same between drug and placebo groups.

Along similar lines, if BOLD signal changes in response to longer-term drug treatment are predictive of clinical improvement (e.g. Cullen et al., 2016, discussed in Section 4), this suggests that the BOLD signal changes are likely reflecting neurobiological changes that contribute to the observed behavioural changes. Again, confidently ascribing these BOLD changes to neuronal activity changes still depends on excluding the possibility that the drug directly interferes with neurovascular coupling. Bourke and Wall (2015) provide a good review of strategies through which such confounding factors in the interpretation of pharmacological fMRI can be mitigated.

Thus, overcoming the interpretational difficulties involved in comparing BOLD signal differences between pharmacologically treated and untreated groups is not easy. The most promising experimental approach for understanding what between-group BOLD signal differences represent is to simultaneously gain a direct measure of neural activity and/or blood flow. It is equally important to be knowledgeable about the ways in which any drug of interest may interact with neurovascular coupling or energy metabolism directly. As we build a fuller picture of all of the effects that fluoxetine has on the developing brain, we will learn when we need to be particularly cautious about attributing BOLD signal differences to neuronal activity differences, and when neurovascular or metabolic confounds are less of a concern.

## 9 Summary

We have previously investigated how typical neurodevelopment (Harris et al., 2011; see also Church et al., 2012) and autistic neuropathology (Reynell and Harris, 2013) involve changes to the relationship between neural activity, blood flow and brain energy use, which can alter the way that BOLD signal differences in these populations, compared to control groups, should be interpreted. Here, we have extended this investigation to consider the effects that pharmacological antidepressant treatment – particularly during the sensitive neurodevelopmental period of adolescence – have on the physiological basis of the BOLD signal.

Fluoxetine is used to treat depression because it alters the brain in a way that significantly improves the symptoms of depression for a significant proportion of the people who take it (Hetrick et al., 2010). Although not fully understood, the primary mechanism of action is thought to be through inhibition of serotonin transporters (see Section 3). However, in this paper we have described many other pharmacological actions of fluoxetine that would not only alter neuronal activity but may also alter the signaling pathways responsible for producing blood flow responses, independent of neuronal activity, as well as brain energy use.

Despite the significant amount of research into the short- and long-term effects of fluoxetine treatment on various components of the signalling pathways between neurons, glial cells, blood vessels and cellular metabolism, we are far from having a complete picture of how the drug influences the BOLD signal. Nevertheless, being aware of these possible changes in the relationship between neuronal activity and the BOLD response in different groups of interest can help researchers plan their studies in a way that will minimise the risk of incorrectly interpreting fMRI data. For instance, experiments such as those carried out by Tao et al. (2012) minimise the additional complications of comparing across populations by allowing for within-participant comparisons, before and after drug treatment. Most importantly, though, awareness of the possible non-neuronal sources of a BOLD signal difference between two groups can ensure that these differences are not incorrectly attributed to changes in neuronal activity, or vice versa for a BOLD signal similarity.

We believe that fMRI is a vital tool with which to study affective disorders, such as depression, in humans. We also know that the key to gaining meaningful information from these studies is understanding the relationship between neuronal activity, blood flow response and brain oxygen use, and how this relationship is affected by development, pathology and pharmacology. More inter-disciplinary collaboration, with research crossing the boundaries of cognitive, cellular and molecular neuroscience, will be key to achieving this understanding.

## Conflict of interest

None.
